# *In vitro* evaluation of 3D printed polycaprolactone scaffolds with angle-ply architecture for annulus fibrosus tissue engineering

**DOI:** 10.1093/rb/rbz011

**Published:** 2019-04-22

**Authors:** T R Christiani, E Baroncini, J Stanzione, A J Vernengo

**Affiliations:** 1 Department of Biomedical Engineering, Rowan University, 201 Mullica Hill Road, Glassboro, NJ, USA; 2 Department of Chemical Engineering, Rowan University, 201 Mullica Hill Road, Glassboro, NJ, USA

**Keywords:** surface modification, scaffolds, materials structure, 3D printing

## Abstract

Tissue engineering of the annulus fibrosus (AF) is currently being investigated as a treatment for intervertebral disc degeneration, a condition frequently associated with low back pain. The objective of this work was to use 3D printing to generate a novel scaffold for AF repair that mimics the structural and biomechanical properties of the native tissue. Multi-layer scaffolds were fabricated by depositing polycaprolactone struts in opposing angular orientations, replicating the angle-ply arrangement of the native AF tissue. Scaffolds were printed with varied strut diameter and spacing. The constructs were characterized morphologically and by static and dynamic mechanical analyses. Scaffold surfaces were etched with unidirectional grooves and the influence on bovine AF cell metabolic activity, alignment, morphology and protein expression was studied *in vitro*. Overall, the axial compressive and circumferential tensile properties of the scaffolds were found to be in a similar range to the native AF tissue. Confocal microscopy images indicated that cells were able to attach and spread on the smooth polycaprolactone scaffolds, but the surface texture induced cellular alignment and proliferation. Furthermore, immunofluorescence analysis demonstrated the aligned deposition of collagen type I, aggrecan and the AF-specific protein marker tenomodulin on the etched scaffolds. Overall, results demonstrated the potential for using the scaffolds as a template for AF regeneration.

## Introduction

In the USA, reports have shown that low back pain (LBP) affects between 70 and 85% of the population at some point during their lifetime [1]. LBP is frequently associated with degenerated and or injured intervertebral disc (IVD) [2]. The IVD is composed of three basic structures: a central nucleus pulposus (NP), a peripheral annulus fibrosus (AF) and two layers of cartilage covering the top and bottom called cartilage endplates [3]. Changes in the matrix composition of the IVD are associated with LBP [2]. Specifically, disc degeneration can result from damage to or dehydration of the NP, which reduces its hydrostatic pressure on the internal surface of the AF. This causes abnormal compressive stresses on the AF, causing tears, cracks and fissures after repeated loads. Back pain can develop as a result of NP tissue migrating through the AF, called a herniation, and impinging on nerve roots [4, 5]. Currently, disc degeneration may be treated pharmacologically, with physical therapy, or surgical intervention. However, these treatments do not address the underlying degenerative process, so recurrent symptoms and re-operation is common [6–8].

Given these drawbacks, biologic-based therapies, such as tissue engineering, are currently being investigated for repair of the damaged AF to prevent recurrent herniation. However, the development of such strategies is a significant challenge due to the complex hierarchical structure and heterogeneity of the tissue. The AF is a layered structure composed of concentric sheets of Collagen I and II, called lamellae (9). Each adjacent lamella comprised collagen fibers oriented in angles from ±45° in the inner AF to ±30° in the outer AF, gradually decreasing in angle in a linear fashion [9]. The concentric layers form an angle-ply laminate structure [10, 11]. Attempts to repair the AF in pre-clinical studies have been met with moderate success and are summarized in a recent review [12]. Fuller *et al*. [13] studied repair of an AF lesion site in an ovine model using an acellular collagen sponge soaked with hyaluronan oligosaccharides. Remodeling was only detected in the outer AF after 6 months. Hegewald *et al*. [14] studied AF repair in an ovine model using a textile polyglycolic acid/polyvinylidene fluoride acellular implant. Directional matrix was deposited throughout the AF defect at 12 weeks, but in provocative pressure testing, no statistical differences were found between repaired and injured groups. In 2003, Sato *et al*. [15] described implantation of allografted AF cells seeded in an atelocollagen honeycomb-shaped scaffold. After 12 weeks, hyaline-like cartilage tissue was produced in the AF defect, but the organization did not resemble that of native AF tissue.

*In vitro*, researchers have made progress in developing scaffolds that replicate the fibrous nature of the AF using electrospinning of polycaprolactone (PCL) [10, 11, 16–18]. Consistent among these studies is the result that oriented alignment of the fibers results in better retention of AF phenotype and the aligned deposition of tissue [11, 19, 20]. Despite progress in the use of electrospinning for AF repair, the method produces close-packed fibers, which may lead to low pore interconnectivity. Furthermore, although electrospinning scaffolds can be layered [16], it is difficult to generate porous, 3D structures, limiting area for cell migration into the material and the deposition of matrix [11, 17].

Interestingly, 3D printing technologies have not been extensively researched for fabrication of IVD scaffolds. However, 3D printing offers great potential for the attainment of angle-ply geometry and direct printing of various cell types, scaffold materials and biomolecules [21]. Rosenzweig *et al.* [22] printed mesh structures of acrylonitrile butadiene and polylactic acid. Proliferation and extracellular matrix (ECM) deposition by NP cells on the scaffolds was demonstrated. Alternatively, resin-based porous scaffolds were printed out of poly(trimethylene carbonate) [23]. The aligned deposition of collagen by human adipose-derived mesenchymal stem cells (MSCs) was shown within the scaffold pores. In another study, polyurethane was printed in concentric rings replicating the lamellar structure of the AF. The materials were shown to be biocompatible with bovine IVD cells, but ECM formation was not evaluated [24].

In this work, we used 3D printing to advance AF tissue regeneration by generating a scaffold with angle-ply architecture, requisite mechanical properties and uniform cell attachment. Addressing these points, we printed laminar constructs comprised PCL struts oriented at alternating angles of ±30°. The first objective of our work was based on a 2 ×2 factorial design to vary scaffold architecture. The diameter of the PCL struts and spacing between the struts were varied in order to engineer a construct with suitable morphological and mechanical properties for AF repair. To this end, the scaffolds were assessed by scanning electron microscopy (SEM) and by static and dynamic mechanical analyses (DMA). The second objective of our work focused on the isolation and culture of bovine AF cells on the laminar construct. A unidirectional surface texture was introduced onto the PCL constructs, and the effects on seeded bovine AF cell metabolic activity, alignment, morphology and protein expression was studied over 21 days *in vitro*. The results obtained from this investigation will provide a foundation for further studies on the use of 3D printing and develop scaffolds that can induce functional assembly of ECM mimicking the native AF.

## Materials and methods

### Materials

PCL (Mn = 72, 918 Da) filament with a 1.75-mm diameter was purchased from Tipeye (part no. OD3DLINE-US-6PCL05). Dulbecco’s phosphate buffered saline (PBS), fetal bovine serum (FBS) and trypsin–ethylenediaminetetraacetic were purchased from VWR Life Science. High glucose Dulbecco’s minimum essential medium (DMEM), GlutaMAX™, Pyruvate Supplement and Antibiotic-Antimycotic 100× were purchased from Gibco™. Triton X-100 and Tris buffered saline (TBS, 10×, pH 7.4) were purchased from Fisher Scientific. Ascorbic acid, Type I collagen from calf skin, 1-ethyl-(3-3-dimethylaminopropyl) carbodiimide hydrochloride (EDC), and Collagenase P were purchased from Sigma Aldrich.

### Scaffold fabrication

**Figure 1:**
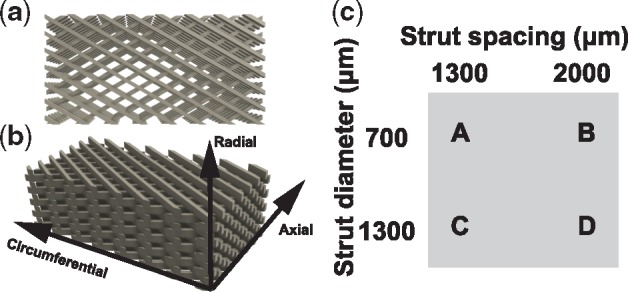
Top view (**a**) and side view (**b**) of the PCL scaffold mimicking the angle-ply structure of the AF. (**c**) Factorial study designed to investigate the properties of the PCL scaffolds

Models (Fig. 1a) were made as standard tessellation language files using the cloud-based 3D CAD system OnShape. Constructs were printed on a Robo 3D R1 (Robo, San Diego, CA, USA). PCL was dispensed through a 400-μm nozzle size, maintained at 130°C at a deposition rate of 340 mm/min onto a bed maintained at 40°C. Scaffolds were drawn with two different strut thicknesses (700 and 1300 μm) and two different strut spaces (1300 and 2000 μm) (Fig. 1b). The constructs were fabricated as rectangular shaped (24 L ×48 mm W) and comprised four total layers, each with thickness set to 1000 μm. The PCL struts in each layer were oriented at angle theta (*θ*) of ±30°. After printing, smaller rectangular samples were excised for porosity and SEM analysis (9 L ×6 mm W), DMA (45 L×9 mm W), tensile testing (17 L×5 mm W), compressive testing (8 L×5 mm W) or cell culture (5 L×3 mm W). For cell culture, a group of PCL scaffolds were manually surface-modified with unidirectional grooves using 30 μm grit sandpaper and designated as the ‘etched’ group. PCL scaffolds that did not receive a surface treatment and used for cell culture are designated as the ‘smooth’ group.

### Porosity measurement

The porosity (*P*) was experimentally measured by analyzing the mass and volume of the samples (*n* = 5), as (25): where *ρ*_PCL_ is the density of solid PCL after printing, measured experimentally to be 1.13 g/cm^3^.

(1) $$P=1-\left(\frac{M_{S}}{V_{S}}\cdot \frac{1}{\rho_{\mathrm{PCL}}}\right),$$

where *M*_S_ is the scaffold’s mass, *V*_S_ is the scaffold’s volume and *ρ*_PCL_ is the density of solid PCL after printing, measured experimentally to be 1.13 g/cm^3^.

### Scanning electron microscopy

Smooth and etched scaffold samples were sputtered in gold and characterized by SEM (Phenom Pure, Phenom-World B.V., The Netherlands). Topographical feature size was quantified by ImageJ (NIH, Bethesda, MD, USA).

### Mechanical characterization

#### Static mechanical analysis

Samples were loaded onto an EZ-X Series Universal Electromechanical test frame (Shimadzu, Kyoto, Japan), subjected to a pre-load of 0.1 N, and stretched or compressed at room temperature at a rate of 0.2% strain/s. For tensile testing, samples (*n* = 5) were clamped with serrated grips and stretched in the circumferential tensile direction. For compressive testing, samples (*n* = 5) were loaded unconfined in the axial direction. The compressive and tensile moduli were computed by converting load–displacement data to stress–strain and measuring the slope of the chord drawn between 5 and 15% strain.

#### Dynamic mechanical analysis

Samples (*n* = 3) were loaded onto a Q800 DMA (TA Instruments, New Castle, DE, USA) for single cantilever analysis using a 1 Hz frequency 7.5 μm amplitude and 0.35 Poisson’s ratio. Samples were equilibrated at −80°C for 10 min then ramped to 40°C at a rate of 2°C/min. Values of storage modulus (*E*′), loss modulus (*E*″), mechanical loss (tan *δ*) and complex modulus (E*) were recorded over the temperature range studied. The glass transition temperature (*T*_g_) was determined as the temperature at which tan *δ* peaked. The molecular weight between entanglements (*M*_e_) was calculated from the rubbery storage modulus above *T*_g_ as:

(2) $$E'=\frac{3\;\rho \;R\;T}{M_{e}}$$

where *ρ* is the density of PCL, *R* is the universal gas constant, *T* is the temperature, in this case 25°C, and *E′* is the storage modulus at 25°C.

### Cell culture studies

#### Collagen coating

To improve the biological properties of synthetic PCL [26–28], the smooth and etched scaffolds were coated in collagen. Excised scaffolds for cell culture were soaked in 0.5% (w/v) solution of Type I collagen in 0.05 M acetic acid at room temperature [26] for 1 h. Subsequently, the scaffolds were allowed to dry in air for 24 h, followed by immersion in a 50 mM EDC solution in 95% ethanol for 24 h at room temperature, to induce crosslinking of the collagen layer [27, 28]. Post-crosslinking, the scaffolds were washed five times in deionized water and lyophilized. Immediately prior to cell culture, scaffolds were sterilized by immersion in 70% ethanol.

#### Cell isolation

Caudal IVDs were aseptically excised from bovine tails within 24 h of death. Cartilage endplate were removed with a razor blade and the outer AF was dissected with a 1.5 mm biopsy punch. The tissue samples were placed in culture medium, composed of DMEM supplemented with 10% FBS, 100 IU/ml penicillin–100 μg/ml streptomycin, 0.25 μg/ml amphotericin B and 50 µg/ml ascorbic acid, supplemented with 0.5% (w/v) collagenase P overnight at 37°C. The resulting suspension was passed through a 100 µm cell strainer, pelleted, washed in sterile PBS and re-suspended in the same medium without the addition of collagenase P. Cellular viability and number were verified and counted using the trypan blue exclusion method. For expansion, the bovine AF cells were seeded in monolayers at a density of 2× 10^4^ cells/cm^2^ and passaged up to P3 to obtain sufficient cell numbers for the experiments.

For cell seeding, 400 μl of AF cell suspension (100 000 cells/ml) was applied over each scaffold. Cells were allowed to attach for 48 h under static culture conditions. At this point, samples were aseptically transferred to new wells and covered in fresh culture medium. Medium was refreshed three times per week for the duration of the culture period.

#### Proliferative activity

Cell metabolic activity (*n* = 5 smooth, *n* = 5 etched) was evaluated using Alamar Blue^®^ (AB, Biorad). Scaffolds were first transferred to new wells to measure the metabolic activity of adhered cells. After 2, 14 and 21 days, cell culture medium containing 10% (v/v) AB reagent was added to each well and the plates were incubated for 5 h, after which 200 µl of each solution was transferred to white opaque 96-well plates in triplicate. Absorbance was measured at 570/600 nm, using a microplate reader (Spectra Mac M3, Molecular Devices). Percent reagent reduction was calculated according to the manufacturer’s instructions by normalization to the mean absorbance of blank controls (media with no cells) and compared at each time point as an indirect measure of proliferative activity.

#### Cell morphology

At 21 days, smooth and etched PCL scaffolds were removed from culture medium, washed in sterile PBS, and fixed with 3.7% formaldehyde in PBS for 10 min at room temperature. Subsequently, samples were washed with PBS again, and frozen before analysis. To evaluate cell attachment and morphology, scaffolds (*n* = 3 per group) were immersed in 0.1% (v/v) Triton X-100 in PBS for 5 min at room temperature, washed with PBS, stained with Alexa Fluor^®^ 647 (Invitrogen) according to the manufacturer’s instructions, and counterstained with 0.5 µg/ml ethidium bromide. These particular fluorescent markers were chosen because the PCL scaffolds do not auto fluoresce at the excitation and emission maxima.

#### Immunofluorescent analysis of protein expression

Fixed and frozen scaffold samples were thawed and immersed in 0.3% (v/v) Triton X-100 for 5 min. Subsequently, non-specific binding was blocked by incubation at room temperature in 10% (v/v) goat serum in 1× TBS. Scaffolds were incubated at room temperature for 1 h in mouse monoclonal antibody against bovine collagen type I (COL1, Abcam, 1:100 dilution), collagen type II (COL2, Abcam, 1:200 dilution), aggrecan (ACAN, Abcam, 1:50 dilution) or rabbit anti-bovine polyclonal antibody against tenomodulin (TNMD, Fisher Scientific, 1:100 dilution). Following three rinses in 1× TBS, scaffolds were treated with Alexa Fluor^®^ 647 secondary antibodies (1:200 dilution of goat anti-mouse or goat anti-rabbit, Abcam) for 30 min at room temperature. Staining without incubation of the primary antibody was conducted to ensure specificity of the secondary antibody. Subsequently, all scaffolds were washed three times in 1× TBS, counterstained with 0.5 µg/ml ethidium bromide for 30 min at room temperature, and imaged using a confocal microscope (Model A1+, Nikon Instruments Inc., Melville, NY, USA) under 20× magnification. During image processing, cell nuclei were modified to display in cyan for enhanced contrast.

### Statistical analysis

Quantitative data sets were subjected to an analysis of variance and unpaired two-sample *t* tests, assuming equal variance, with a post hoc Bonferroni correction factor and statistical significance was set at the 95% confidence interval (*α* =0.05).

## Results

### Scaffold morphological features

**Figure 2:**
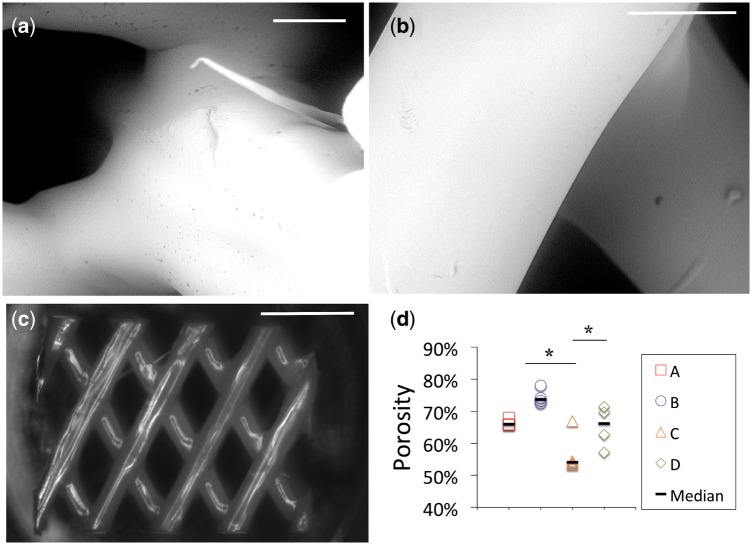
Morphological evaluation of the 3D printed PCL scaffolds. (**a–c**) Representative SEM images of scaffold A. (**d**) Increased spacing and decreased strut diameter resulted in increasing trends in porosity. The dashes show the median difference. Scale bars (a) 300 μm, (b) 500 μm, (c) 3000 μm.*Samples that were statistically significant, *P* < 0.05

The 3D scaffolds were designed as shown in Fig. 1a and printed with the size parameters shown in Fig. 1b. The morphology observed with SEM (Fig. 2a–c) revealed a homogenous porous structure and the ability of the design to mimic the angle-ply architecture of the AF. Further SEM analysis revealed that the surface morphology of smooth and etched PCL did not change after collagen coating (data not shown). The median measured porosities for scaffolds ‘A’ through ‘D’ ranged from 54 to 74%. Porosity values increased with increased strut spacing and decreased strut diameter (Fig. 2d).

### Scaffold mechanical properties

**Figure 3:**
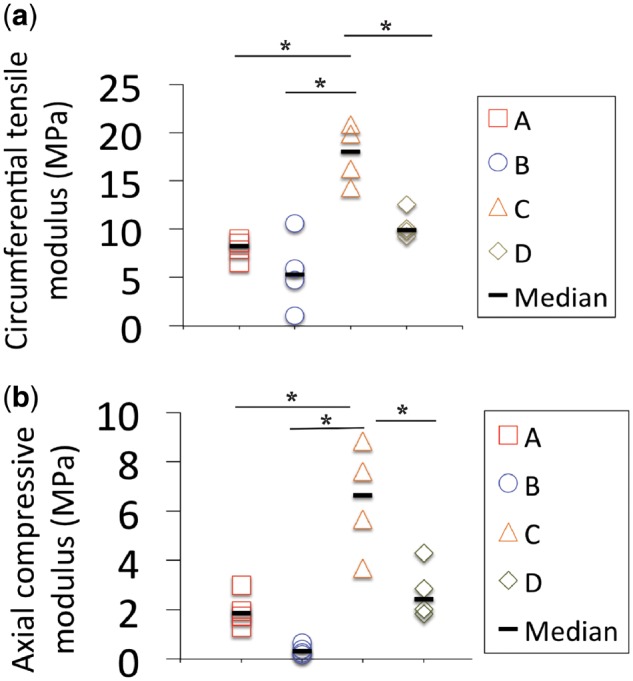
Static mechanical properties of PCL scaffolds a through D. (**a**) Unconfined compressive moduli measured in the axial direction. (**b**) Circumferential tensile moduli. Mechanical properties increased with strut diameter and decreased strut spacing. The dashes show the median difference. *Samples that were statistically significant, *P* < 0.05

The static mechanical properties of the scaffolds were evaluated at room temperature under circumferential tension and axial compression. Overall, scaffolds with lower porosity exhibited higher mechanical properties. Median tensile moduli (Fig. 3a) ranged from 5.3 ± 3.1 MPa (‘B’) to 18.0 ± 3.1 MPa (‘C’). With strut spacing held constant at 1200 μm, increasing strut diameter from 700 μm (‘A’) to 1300 μm (‘C’) resulted in a significant increase (*P* < 0.05) in tensile modulus from 8.22 ± 1.0 to 18.02 ± 3.1 MPa. With strut diameter held constant, decreasing the spacing from 2000 μm (‘C’) to 1300 μm (‘D’) resulted in a significant increase (*P* < 0.05) in tensile modulus from 9.92 ± 1.8 to 18.02 ± 3.1 MPa. The median compressive moduli (Fig. 3b) ranged from 0.31 ± 0.2 MPa (‘B’) to 2.42 ± 1.1 MPa (‘C’). Similarly to trends in tensile modulus, increasing strut diameter (‘A’ versus ‘C’) and decreasing strut spacing (‘C’ versus ‘D’) resulted in statistically significant increases in compressive moduli values (*P* < 0.05).

**Figure 4:**
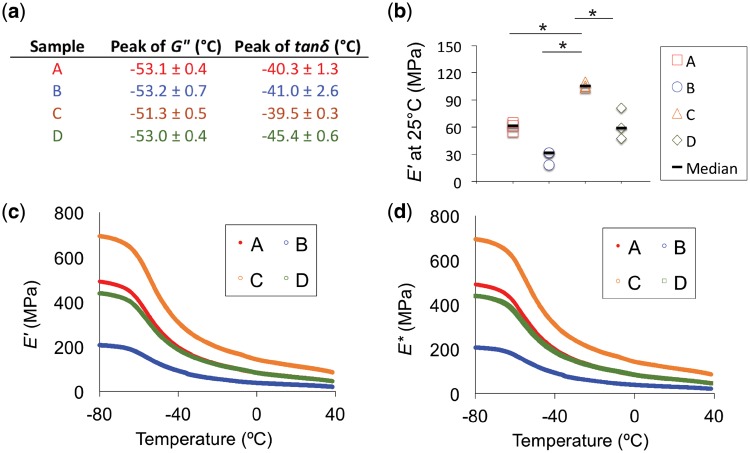
Single cantilever dynamic mechanical analysis was performed at a constant frequency of 1 Hz. Evolution of the storage modulus (*E*′), loss modulus (*E*″), mechanical loss (tan *δ*) and complex modulus (E*) with temperature was recorded. (**a**) *T*_g_ values did not vary significantly across the design matrix. (**b**) *G*′ values at 25°C for scaffolds A–D showed the same trends as the static mechanical properties. The dashes show the median difference. (**c**, **d**) Mean values for *E*′ and E* show that *E*′ is primarily governed by E* over the temperature range studied. *Samples that were statistically significant, *P* < 0.05

DMA analysis confirmed that *T*_g_ values for the scaffolds (Fig. 4a) did not vary across the design matrix, an indication of the consistency of the printing conditions with respect to the molecular structure produced. The median values for storage moduli (*E*′) at 25°C, reported in Fig. 4b, ranged from 31.59 ± 7.7 to 105.60 ± 3.3 MPa. *E*′ is a quantitative measure of a material’s elasticity, or capability to store energy and resist permanent deformation. Consistent with values for the static moduli, *E*′ values trended toward increasing with decreasing strut spacing and increasing strut diameter. Increasing strut diameter (‘A’ versus ‘C’) and decreasing strut spacing (‘C’ versus ‘D’) resulted in statistically significant increases in *E*′ values (*P* < 0.05). This trend was consistent over the entire temperature range studied (Fig. 4c). The complex modulus (E*, Fig. 4d) is a function of both the *E*′ and the loss modulus, *E*″. *E*″ is a quantitative measure of a material’s viscous behavior, or propensity to dissipate energy. A comparison between *E*′ and E* (Fig. 4c and d) reveals that the PCL scaffolds exhibited a significant elastic response, since the values for E* are predominated by *E′* [24]. *M*_e_ is the measure of the molecular weight of chain segments between nodes of the interacting polymer chains. Though *M*_e_ characterizes microscopic polymer interactions, the tested scaffolds contained varying macroscopic gaps due to varying strut diameter and spacing. Consequently, although all were made of the same PCL material, the *M*_e_ values of the scaffolds differed from that of the solid PCL and from each other. In general, scaffolds with smaller strut diameter, larger strut spacing and high porosity exhibited higher *M*_e_ and lower *E*′. Conversely, increased strut diameter, decreased strut spacing and low porosity gave rise to lower *M*_e_ and higher *E*′. Therefore, larger interstitial spaces led to weaker scaffold mechanical properties. Summarized properties of scaffold architecture, porosity, molecular weight between entanglements and storage modulus for each scaffold can be found in Table 1.

**Table 1:** Summary of the mechanical properties for each of the investigated PCL constructs as well as solid PCL

Sample	Strut diameter (µm)	Strut spacing (µm)	Porosity (%)	Molecular weight between entanglements (*M*_e_) at 25°C (g/mol)	***E***′ **at 25°C (MPa)**
**A**	700	1300	66.0±1.0	132.4±7.6	60.3±5.1
**B**	700	2000	73.7±2.4	309.2±81.1	27.2±7.7
**C**	1300	1300	54.1±6.6	72.4±1.2	106.3±3.3
**D**	1300	2000	66.2±6.5	137.0±3.3	62.4±17.1
**Solid**				19.0±0.4	1678.44±62.8

### Scaffold biological properties

**Figure 5:**
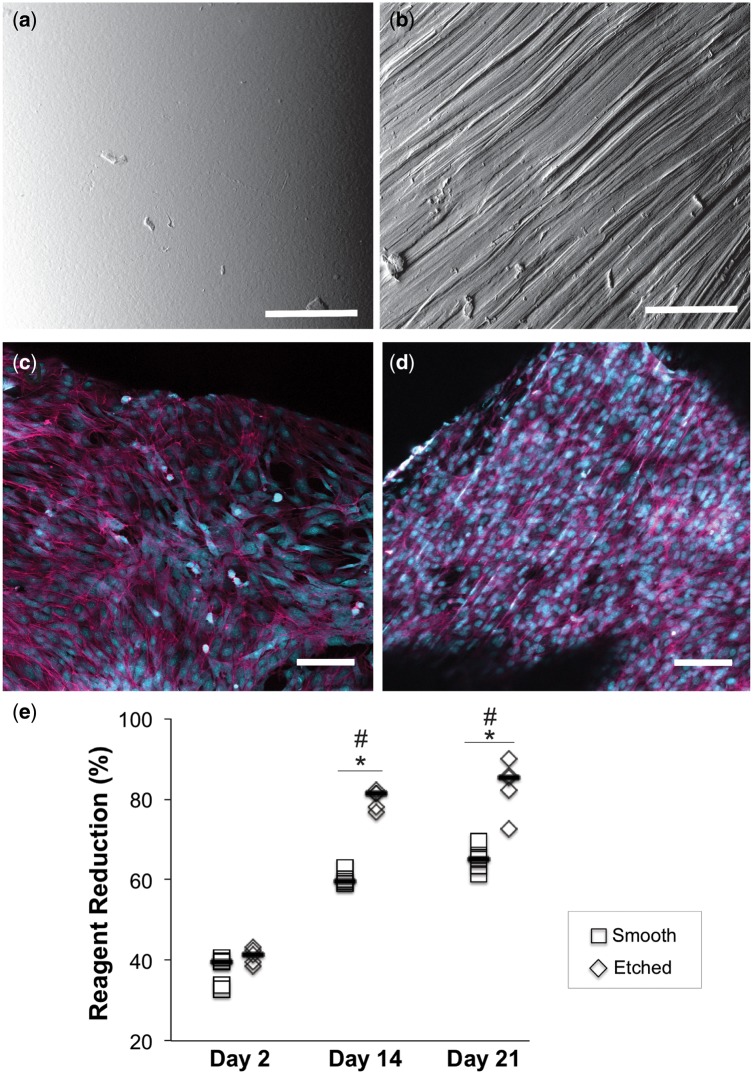
SEM micrographs of scaffold C showing (**a**) smooth surface texture after printing and (**b**) unidirectional channels etched using 30 μm particle size sandpaper. Bovine AF cells were cultured *in vitro* on both types of surfaces for 21 days, stained with Alexa Fluor^®^ 647 phalloidin (magenta) and counterstained with ethidium bromide (nuclei are shown in cyan to enhance contrast). (**c**) Cells cultured on smooth PCL demonstrated random alignment. (**d**) Cells cultured on etched PCL had a tendency to align along the underlying surface texture. (**e**) Alamar blue assay showed significant increases metabolic activity over the culture period, confirming cell proliferation. Scale bars=100 μm. *Statistical significance at single time point; ^#^statistical significance compared with Day 2*, P *<* *0.05

Due to its high mechanical properties, scaffold ‘C’ was chosen for evaluation in the cell culture studies. The biological properties of scaffold ‘C’ were investigated as a function of surface texture. Directly after printing, scaffold ‘C’ exhibited a very smooth surface (Fig. 5a). Manual etching by the unidirectional application of 30 μm grit sandpaper across the PCL surface (back and forth several times) successfully resulted in the formation of parallel grooves (Fig. 5b). The size of the resulting topographical features ranged from 2 to 12 μm. As visualized by the cytoskeletal staining with phalloidin, bovine AF cells cultured on smooth surfaces exhibited attached, spindle-like morphology and spread randomly across the surface by Day 21 (Fig. 5c). In contrast, the culture of AF cells on etched surfaces exhibited a greater degree of alignment and a cytoskeletal arrangement coinciding with the underlying surface texture (Fig. 5d).

The metabolic activity of the cells over the 21-day culture period was quantitated with an Alamar blue (AB) assay (Fig. 5e). Cells cultured on both types of surfaces exhibited statistically significant increases in AB reagent reduction at Days 14 and 21 relative to Day 2 (*P* < 0.05), indicative of the PCL surfaces ability to support cell proliferation. At Days 14 and 21, AB reduction was significantly higher (*P* < 0.05) for the cells cultures on etched versus smooth surfaces.

**Figure 6:**
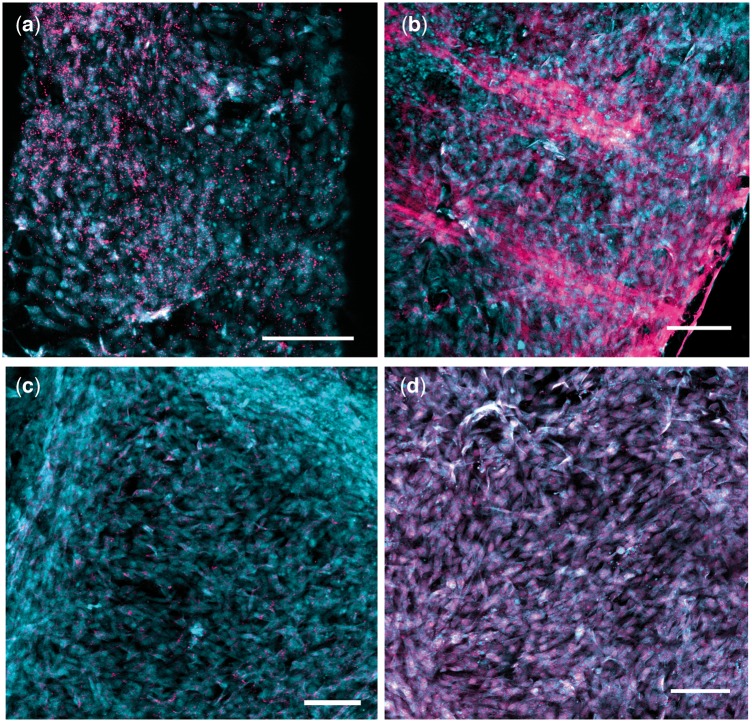
Immunocytochemical results at Day 21 of culture for bovine AF cells seeded on smooth PCL surfaces. Scaffolds were treated with primary antibodies targeting (**a**) ACAN, (**b**) COL 1, (**c**) COL 2, (**d**) TNMD. Alexa Fluor^®^ 647 was used for secondary antibody, and cells were counterstained with ethidium bromide. Cell nuclei are shown in cyan to enhance contrast. Scale bars=100 μm

**Figure 7:**
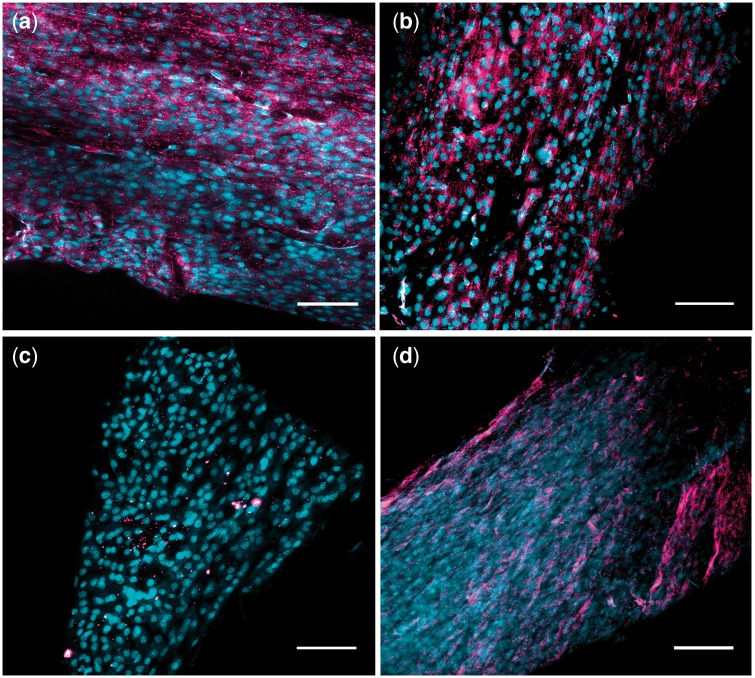
Immunocytochemical results at Day 21 of culture for bovine AF cells seeded on smooth PCL surfaces. Scaffolds were treated with primary antibodies targeting (**a**) ACAN, (**b**) COL 1, (**c**) COL 2, (**d**) TNMD. Alexa Fluor^®^ 647 was used for secondary antibody and cells were counterstained with ethidium bromide. Cell nuclei are shown in cyan to enhance contrast. Scale bars=100 μm

Immunocytochemical staining was used to detect the presence or absence of known IVD ECM components, COL1 (Fig. 6a), COL2 (Fig. 6b) and ACAN (Fig. 6c) [29–40]. Additionally, we evaluated the presence of an AF-specific marker TNMD (Fig. 6d) [41]. Significant positive immunolabeling for COL1, ACAN and TNMD for cells cultured on the PCL surfaces can be identified by the magenta color (Fig. 6). Minimal positive staining for COL2 was detected. This result was expected, since the cells were extracted from the outer AF tissue, which is predominantly Type I collagen rather than Type II [42]. Immunocytochemical staining for the etched surfaces detected the same strong positive expression of COL1, ACAN and TNMD and weak expression of COL2. Importantly, alignment of the proteins with the underlying surface texture can be observed (Fig. 7).

## Discussion

Current strategies for AF repair demonstrated modest success *in vivo*, but do not completely restore the native structure and function to the degenerated IVD. Thus, there is a clinical need for new solutions for AF regeneration. In this study, we describe the fabrication and characterization of 3D printed PCL scaffolds for AF tissue engineering, which possess angle-ply architecture and exhibit several promising properties for AF repair.

Biomechanical performance is critical for an AF repair device because it is necessary for withstanding *in vivo* stress concentrations, mitigating or preventing further degeneration after implantation and protecting seeded cells. The recommended tensile modulus for an AF repair material is 11–29 MPa in the circumferential direction [43–45]. Scaffold ‘C’, with a median tensile modulus of 18 MPa, coincided with this recommended range, while ‘A’, ‘B’ and ‘D’ were lower, but similar in magnitude. Furthermore, the maximum tensile strain in the posterior AF was reported as 65% [46, 47]. All the tested PCL formulations proved to be extremely ductile and far exceeded the strain level of 65% without failure (data not shown). Native AF compressive modulus has been reported to be ∼200 kPa [48]. All the scaffolds produced for this study exceeded this mechanical requirement. Furthermore, DMA analysis revealed appreciable elastic behavior for scaffolds ‘A’ through ‘D’, indicating the potential of the materials to resist permanent deformation with loading in the disc. More specifically, scaffold ‘C’ had the largest strut diameter and smallest spacing, thus resulting in the lowest porosity, lowest *M*_e_ and highest *E*′. Scaffold architecture must be considered when optimizing porosity and mechanical properties to allow for cell proliferation, aligned deposition of ECM and endurance of *in vivo* loading.

The fluorescence imaging and AB results confirm the biocompatibility of the PCL scaffolds and corroborate the results of other prior studies demonstrating the promise of PCL in cellular tissue engineering [17, 18, 49–51]. Furthermore, confirmation of COL1, COL2, ACAN and TNMD expression at the protein level demonstrated potential of the PCL to retain AF phenotype. However, a requisite for engineering tissue that mimics the native AF is the deposition of aligned matrix in an angle-ply configuration. Unidirectional surface texture was incorporated onto the PCL struts, based on the hypothesis that the cells would align and deposit matrix along the surface texture. Consistent with our hypothesis, the patterning of the surface induced cellular alignment and the subsequent deposition of aligned proteins. In addition, the etched surfaces resulted in significantly higher AB reagent reduction compared with smooth surfaces at Days 14 and 21. The increase is likely attributable to the increased surface area, allowing for greater cell level of cell proliferation. In this study, the grooves were generated with a range of sizes by passing the sandpaper over the surface multiple times. Future studies should examine the optimum size of the surface features.

PCL is widely known as a relatively stable polymer under hydrolytic degradation, requiring 2–4 years for complete degradation, depending on the molecular weight [52] and morphology [53]. The slow degradation rate makes it promising for the IVD, a load-bearing site with regenerative processes that take place over the long-term [54–57]. Potentially, extrusion during printing can cause degradation or chemical modifications to the PCL chains that impact degradation behavior. In our case, strut diameter and overall porosity can also affect scaffold degradation characteristics. Domingos *et al*. [58] reported that the thermal extrusion process had no effect on molecular weight (*M*_n_), polydispersity index (*M*_w_/*M*_n_) or crystalline fraction of the PCL. Dong *et al*. [59] studied the degradation behavior of polyglycolide (PGA), poly(dl-lactide-co-glycolide) (PLGA) and poly(l-lactide-co-ε-caprolactone) [P(LLA-CL)] with and without cell culture. Cell culture significantly increased the degradation rate of PGA nanofibers, whereas the effect on PLGA and P(LLA-CL) nanofibers was limited. Zhang *et al*. [60] studied the mechanical characteristics of a 3D printed PCL scaffold in an *in vivo rabbit* model. At 12 and 24 weeks post-implantation, the tensile and compressive characteristics were higher for cell-seeded scaffolds than cell-free, attributable to tissue deposition within the scaffold by the cells. Prior to *in vivo* application of this scaffold, in-depth study of the degradation rate as a function of morphology, printing parameters and cell growth will be required.

This is the first report in the literature with a 3D printed AF scaffold that meets the requisite tensile and compressive mechanical requirements and promotes the directional deposition of tissue in an angle-ply configuration. The concept has significant potential for a clinical impact. The 3D printing techniques, combined with clinical imaging, offer the opportunity to create patient-specific implants for the IVD. While the focus of this work is on developing a biomaterial that will advance regeneration of AF tissue, in the long-term, the strategy can be integrated into a 3D printing approach to whole-disc replacement.

There are some limitations with the current study. While the current scaffold captures the angle-ply configuration of the disc, it does not capture the heterogeneity of the native tissue. For instance, the stiffness of AF tissue significantly varies depending on the location. The outer AF, rich in Collagen I, is stiffer than the inner AF, which has higher levels of aggrecan and Collagen II [42, 61]. Correspondingly, the AF phenotype transitions from fibroblast-like in the outer annulus to chondrocyte-like in the inner annulus [62, 63]. Fortunately, 3D printing is a versatile method that allows for modification of the biomaterial design to incorporate site-specific biochemical cues and mechanical gradients, substantially advancing AF scaffold design. Furthermore, an in-depth biomechanical characterization of the scaffolds is needed, to capture its performance under torsion and fatigue conditions. In terms of scaffold biological performance, engineered tissue should be quantified in terms of proteogylcan and collagen content, as well as ratio of Collagen I and II. Additional AF markers should be evaluated at a gene and protein level. These can include cartilage oligomatrix matrix protein [38], glypican 3 [64], integrin-binding sialoprotein [29], and fibulin 1 [65], Collagen V [66], CD146+ [67]. Lastly, adipose and bone derived MSCs have been demonstrated to have potential in AF repair [11, 68]. MSCs can be obtained with less morbidity than somatic cell types [69] and thus have more clinical significance, and should be evaluated in further studies.

## Conclusions

Multi-layer scaffolds were 3D printed by depositing PCL struts in opposing angular orientations of ±30°, replicating the angle-ply arrangement of the native AF tissue. Scaffolds were printed with strut diameters of 700 or 1300 μm and parallel spacing between the struts of 1300 or 2000 μm. The scaffolds exhibited significant elastic responses. The circumferential tensile moduli were of similar magnitude to native AF tissue. The axial compressive properties exceeded what has been reported for native tissue. The scaffold with 1300 μm strut diameter and spacing supported the attachment and proliferation of bovine AF cells, as well as the expression of Collagen I, aggrecan and tenomodulin by the cells. The etching of the scaffold with unidirectional surface texture induced cellular alignment and the deposition of the protein markers along the underlying surface texture. The results of these *in vitro* studies demonstrate the potential for using this novel scaffold in IVD repair.

*Conflict of interest statement*. None declared.
